# Prognostic significance of peripheral and tumor-infiltrating lymphocytes in newly diagnosed stage III/IV non-small-cell lung cancer

**DOI:** 10.3389/fmed.2024.1349178

**Published:** 2024-05-22

**Authors:** Fenge Li, Chong Tian, Yupeng Wang, Huancheng Wu, Mengli Jin, Xueming Du, Jin Yan, Xueling Yang, Haipeng Yu

**Affiliations:** ^1^Department of Oncology, Tianjin Beichen Hospital, Tianjin, China; ^2^Core Laboratory, Tianjin Beichen Hospital, Tianjin, China; ^3^Department of Interventional Therapy, Tianjin Medical University Institute and Hospital, National Clinical Research Center for Cancer, Tianjin, China; ^4^Tianjin’s Clinical Research Center for Cancer, Tianjin Medical University Institute and Hospital, Tianjin, China; ^5^Key Laboratory of Cancer Prevention and Therapy, Tianjin Medical University Institute and Hospital, Tianjin, China; ^6^Department of Neurosurgery, Tianjin Beichen Hospital, Tianjin, China

**Keywords:** tumor-infiltrating lymphocytes, lung cancer, survival, peripheral lymphocytes, newly diagnosed

## Abstract

**Background and aim:**

Lymphocytes are effector cells that fight cancer by killing tumor cells. Here, we aim to explore the prognostic significance of both peripheral and tumor-infiltrating lymphocytes (TILs) in newly diagnosed stage III/IV non-small-cell lung cancer (NSCLC).

**Materials and methods:**

In total, 105 cases of newly diagnosed stage III/IV NSCLC from July 2017 to October 2022 at the Tianjin Beichen Hospital were retrospectively investigated. Peripheral blood samples at the time of diagnosis and tumor tissue slices from these patients were collected. General peripheral blood cell composition and TILs were measured and analyzed via an automatic blood analyzer and immunofluorescence staining analysis. The overall survival (OS) time of all patients was also obtained and analyzed.

**Results:**

The median overall survival (mOS) of all patients is 12 months. The 1-, 2-, and 3-year overall survival rates were 60.5, 28.4, and 18.6%, respectively. Peripheral lymphocyte and neutrophil percentages, serum C-reactive protein (CRP) expression, tumor size, and tumor pathology are the prognostic factors of OS for newly diagnosed stage III/IV NSCLC patients. Moreover, patients with high tumor CD4+ and CD8+ T cell infiltration survived significantly longer compared to patients with low tumor CD4+ and CD8+ T cell infiltration (*p* < 0.0001 and *p* = 0.011, respectively). Compared to low tumor CD33+ cell infiltration, high tumor CD33+ cell infiltration was associated with worse OS (*p* = 0.018). High tumor CD8+ T cell infiltration was associated with lower peripheral lymphocyte number, lower serum CRP expression, smaller tumor size, and better tumor pathology (*p* = 0.012, *p* = 0.040, *p* = 0.012, and *p* = 0.029, respectively).

**Conclusion:**

Increased numbers of peripheral lymphocytes, CD33+ cells, CD4+ TILs, and CD8+ TILs were significantly associated with OS in newly diagnosed stage III/IV NSCLC patients, which were positively associated with several basic clinical factors.

## Introduction

The immune system can effectively defeat bacteria, viruses, and other harmful pathogens that infect the body. It is also the main natural “weapon” against tumor cells which makes immunotherapy increasingly important for cancer treatment (1–3). Lymphocytes are a key component of the immune system. They can be peripherally located, or locally located inside the tumors, which make them tumor-infiltrating lymphocytes (TILs). Moreover, lymphocytes have been manipulated or molecularly targeted in immunotherapy for the treatment of cancer, including TIL therapy, Chimeric Antigen Receptor T-cell therapy (CAR-T), and TCR-T (T Cell Receptor T) therapy (4–8).

TIL therapy has shown positive clinical effects on metastatic melanoma (9), advanced cervical cancer (10, 11), non-small cell lung cancer (NSCLC) (5, 12), colorectal cancer (CRC), head and neck cancer, and ovarian cancer (13, 14). The prognostic role of TILs in a variety of tumors including breast cancer, cholangiocarcinoma, ovarian cancer, and lung cancer has been confirmed and entered into clinical guidelines (12, 15–18). Although systematic meta-analysis and previous studies have reported on the prognostic role of TILs in NSCLC, the clinical baseline of patients from these studies was dispersive and inconsistent, and high-quality larger population studies are needed to confirm the role of TILs and their subtypes in prognosis in specific groups of NSCLC patients (19–21). Furthermore, the prognostic and predictive significance of peripheral lymphocytes and TILs in newly diagnosed stage III/IV NSCLC has not been reported. It is crucial to investigate the association of non-intervened TILs and peripheral lymphocytes, which can be a prognostic predictor for the overall survival of patients via peripheral blood analysis. Furthermore, myeloid-derived suppressor cells (MDSCs), which gradually accumulate in tumor tissues, are a heterogeneous group of myeloid cells with potent anti-immune activity. MDSCs interact with adaptive and innate immune cells and play a key role in negatively regulating the immune response to tumors (22). In the present study, we aim to evaluate the prognostic significance of both peripheral and tumor-infiltrating lymphocytes (TILs) and MDSCs in newly diagnosed stage III/IV NSCLC patients.

## Materials and methods

### Patients and tumor sample collection

In total, 105 newly diagnosed stage III/IV NSCLC patients from July 2017 to October 2022 at the Tianjin Beichen Hospital (Tianjin, China) were retrospectively investigated in this study. This study conformed to the ethical guidelines of the Declaration of Helsinki and was approved by the Tianjin Anti-Cancer Association and the ethics committee of the Tianjin Beichen Hospital. The ethics review of Beichen Hospital was carried out in the form of in-person meetings, and the ethics certificate can be provided as needed. The inclusion criteria of the studied patients were as follows: (1) diagnosed with NSCLC by tumor core biopsy; (2) had not received any anti-tumor treatments including chemotherapy, radioactive therapy, and immunotherapy at the time of getting tumor biopsy; (3) tumor samples can be obtained after pathology diagnosis, and at least two slides of tumor tissues were available for the study; and (4) immediately after diagnosis, all patients received standard chemotherapy (Paclitaxel, 135–175 mg/m^2^ and Cisplatin 75 mg/m^2^) and ^125^I brachytherapy (SNCP-^125^I) (23) until disease progression according to the NCCN clinical guidelines. Patients who did not meet the criteria mentioned above were excluded. The basic clinical characteristics of the 105 studied patients are shown in Table 1. All patients’ data were collected from the hospital medical records, and patients with incomplete medical records were excluded. Informed consent was obtained from all patients or their dependents for participation in the study.

**Table 1:** Patient clinical and demographic characteristics at baseline.

**Characteristics**	**Mean ± SD (*n* = 105)**	**Range**
Age (year)	68 (median)	42–91
Gender (male/female)	61/44	–
Tumor size (cm)	4.94 ± 2.35	1.10–11.70
Disease stage (III/IV)	46/59	–
PS score (≤2/>2)	105/0	–
CEA (ng/mL)	63.79 ± 151.90	0.56–987.00
CA153 (U/mL)	26.38 ± 43.73	4.40–264.8
CRP (mg/L)	27.01 ± 46.71	0.2–284.25
Pathology (SQ/AD)	35/70	–
Pleural effusion (*Yes/No*)	29/76	–
Overall survival	13.95 ± 9.5	1–42

CEA, carcinoembryonic antigen; CA153, cancer antigen 153; CRP, C Reactive Protein; SQ, Squamous; AD, Adenocarcinoma; PS Score, Performance Status Store.

### Immunofluorescence staining analysis

Tumor samples were collected from 71 out of 105 patients, and immunofluorescence staining was successfully performed on 53 samples. Tumor tissue slides were first baked in a 60°C incubator for 20 min before being subjected to the following steps for dewaxing: (1) the slides were immersed in xylene for 10 min twice; (2) the slides were then immersed in anhydrous ethanol for 5 min; (3) the slides were soaked in 95% ethanol for 5 min; and (4) the slides were immersed in 70% ethanol for 5 min. After dewaxing, the slides were washed with PBS twice for 5 min and were kept wet during the entire process. Next, the slides were heated in 0.01 M sodium citrate buffer solution (pH 6.0) in a 95°C water bath for 10 min for antigen retrieval. The slides were then allowed to return to room temperature and were washed with PBS three times for 5 min, followed by blocking with 5% normal goat serum for 30 min at room temperature. For immunostaining, the slides were incubated with primary antibodies of anti-CD4 (1:200, ab133616, Abcam), anti-CD8 (1:200, ab237710, Abcam), anti-CD11b (1:200, 66,519-1-lg, Proteintech), and anti-CD33 (1,200, 67,135-1-lg, Proteintech) at room temperature for 1 h, and then washed with PBS three times for 5 min. The slides were then incubated with fluorescently labeled secondary antibodies: CoraLite488-conjugated Goat Anti-Rabbit IgG (H + L) (SA00013-2, Proteintech) and CoraLite594-conjugated Goat Anti-Mouse IgG (H + L) (SA00013-3, Proteintech) at room temperature in the dark for 1 h, followed by washing with PBS three times for 5 min. For nuclear staining, the slides were incubated with DAPI for 10 min, then washed with PBS three times for 5 min. Finally, the slides were sealed with a fluorescence protection sealing agent. The slides were imaged using an inverted fluorescent microscope (Ts2-FL, NIKON).

### Peripheral blood cell composition analysis

In 105 patients, 5 mL of peripheral blood samples were obtained before performing tumor biopsy. The blood samples were analyzed using the automatic blood analyzer (BC-7500CS, Mindray, Shenzhen, China) according to the manufacturer’s instructions. The numbers and percentages of total white blood cells, lymphocytes, and neutrophils were calculated.

### Laboratory testing for CEA, CA153, and CRP

The expression level of cancer antigen 153 (CA153) in the peripheral blood serum was determined using the CA153 antigen determination kit (Product number CB25803547). The expression level of serum carcinoembryonic antigen (CEA) was determined through the magnetic particle separation method using a CEA assay kit (20,153,401,962, Wantai Cary, Xiamen, China). Serum CRP expression was determined using immunoturbidimetry.

### Follow-up assessment

All patients were followed from the date of obtaining the tumor biopsy up to March 2023 or up to the time of death. The overall survival time of each patient was collected.

### Statistical analysis

All statistical analyses were performed using GraphPad Prism 8.0 (GraphPad Software, La Jolla, California, United States). Survival curves were calculated using the Kaplan–Meier estimate and survival comparisons between groups were calculated using the log-rank (Mantel-Cox) test. Survival time was calculated from the date of obtaining the tumor biopsy up to March 2023 or up to the time of death. An unpaired *t*-test was used to analyze the statistical significance between the two groups. The cutoff values used for comparative analysis were the median values of each parameter including cell populations and serum markers. A *p*-value less than or equal to 0.05 was considered statistically significant.

## Results

### Patient characteristic and univariate analysis of clinical factors for patient overall survival

**Figure 1:**
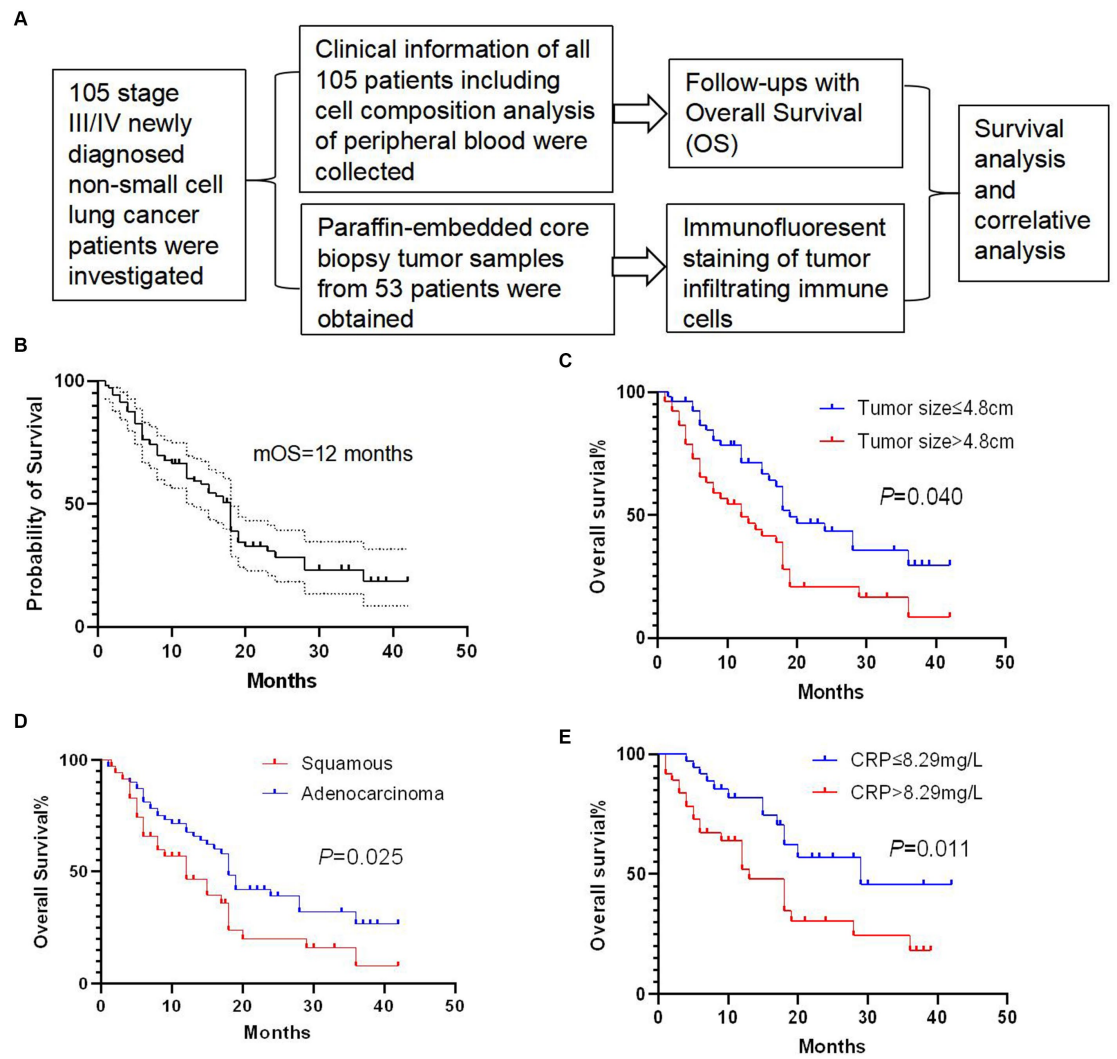
Study and survival analysis of clinical factors. **(A)** Overview of the present study. **(B)** Overall survival (OS) curve of 105 newly diagnosed stage III/IV non-small-cell-lung cancer (NSCLC) patients with a median overall survival (mOS) of 12 months. **(C)** Patients with tumor size below 4.8 cm showed a significantly longer OS than patients with tumor size over 4.8 cm (*p* = 0.040). **(D)** Patients with squamous cell carcinoma experienced a significantly longer OS than patients with adenocarcinoma (*p* = 0.025). **(E)** Patients with a C-reactive protein (CRP) level lower than 8.29 mg/L survived significantly longer OS than patients with a higher CRP level (*p* = 0.011). Survival comparisons between groups were calculated using the log-rank (Mantel-Cox) test.

One hundred and five newly diagnosed stage III/IV NSCLC patients were retrospectively investigated in the present study (Figure 1A). The median overall survival (mOS) of all patients was 12 months, with 1-, 2-, and 3-year overall survival rates of 60.5, 28.4, and 18.6%, respectively (Figure 1B). We performed univariate analysis to explore the prognostic factors in these patients and found that tumor size (Figure 1C), tumor pathology (Figure 1D), and CRP level (Figure 1E) were associated with the overall survival of these patients (Supplementary Table S1). In brief, patients with tumor size below 4.8 cm showed a significantly longer OS than patients with tumor size over 4.8 cm (*p* = 0.040). Patients with squamous lung cancer experienced a significantly longer OS than patients with adenocarcinoma lung cancer (*p* = 0.025). Moreover, patients with a lower CRP level of less than 8.29 mg/L survived significantly longer OS than patients with a higher CRP level (*p* = 0.011). Meanwhile, we found that there was no survival difference in terms of white blood cell count, CA153 expression, peripheral neutrophils count to lymphocyte count, CEA expression, presence of distant metastasis, and presence of pleural effusion (Supplementary Figure S1).

### Patients with higher numbers of CD8+ and CD4+ TIL experienced a significantly longer overall survival

**Figure 2:**
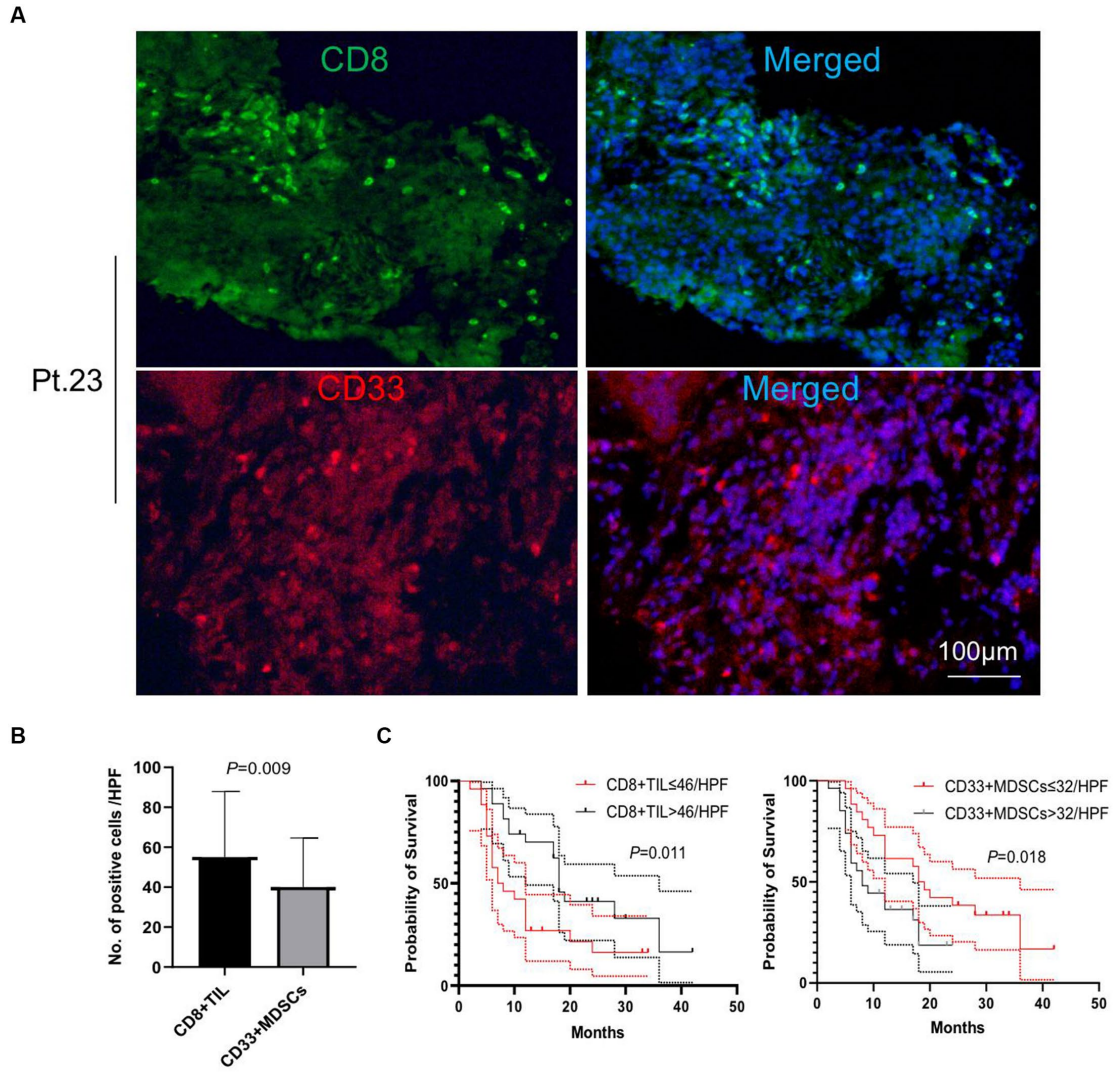
Analysis of tumor-infiltrating CD8+ T cells and CD33+ cells. **(A)** Representative images of immunofluorescence staining of CD8+ T tumor-infiltrating lymphocytes (TILs) and tumor-infiltrating CD33+ myeloid-derived suppressor cells (MDSCs) (CD8 in green, CD33 in red, DAPI in blue). **(B)** Quantitative analysis of CD8+ TILs and tumor-infiltrating CD33+ MDSCs showing that the number of CD8+ TILs was significantly higher than that of the tumor-infiltrating CD33 + MDSCs (*p* = 0.009). **(C)** Patients with CD8+ TILs higher than 46 per HPF and patients with tumor-infiltrating CD33+ MDSCs lower than 32 per HPF showed a significantly longer overall survival compared to patients with lower than 46 CD8+ TILs per HPF or higher than 32 tumor-infiltrating CD33+ MDSCs per HPF (*p* = 0.011 and *p* = 0.018, respectively). HPF, high power field. Survival comparisons between groups were calculated using the log-rank (Mantel-Cox) test. An unpaired *t*-test was used to analyze the statistical significance between the two groups.

**Figure 3:**
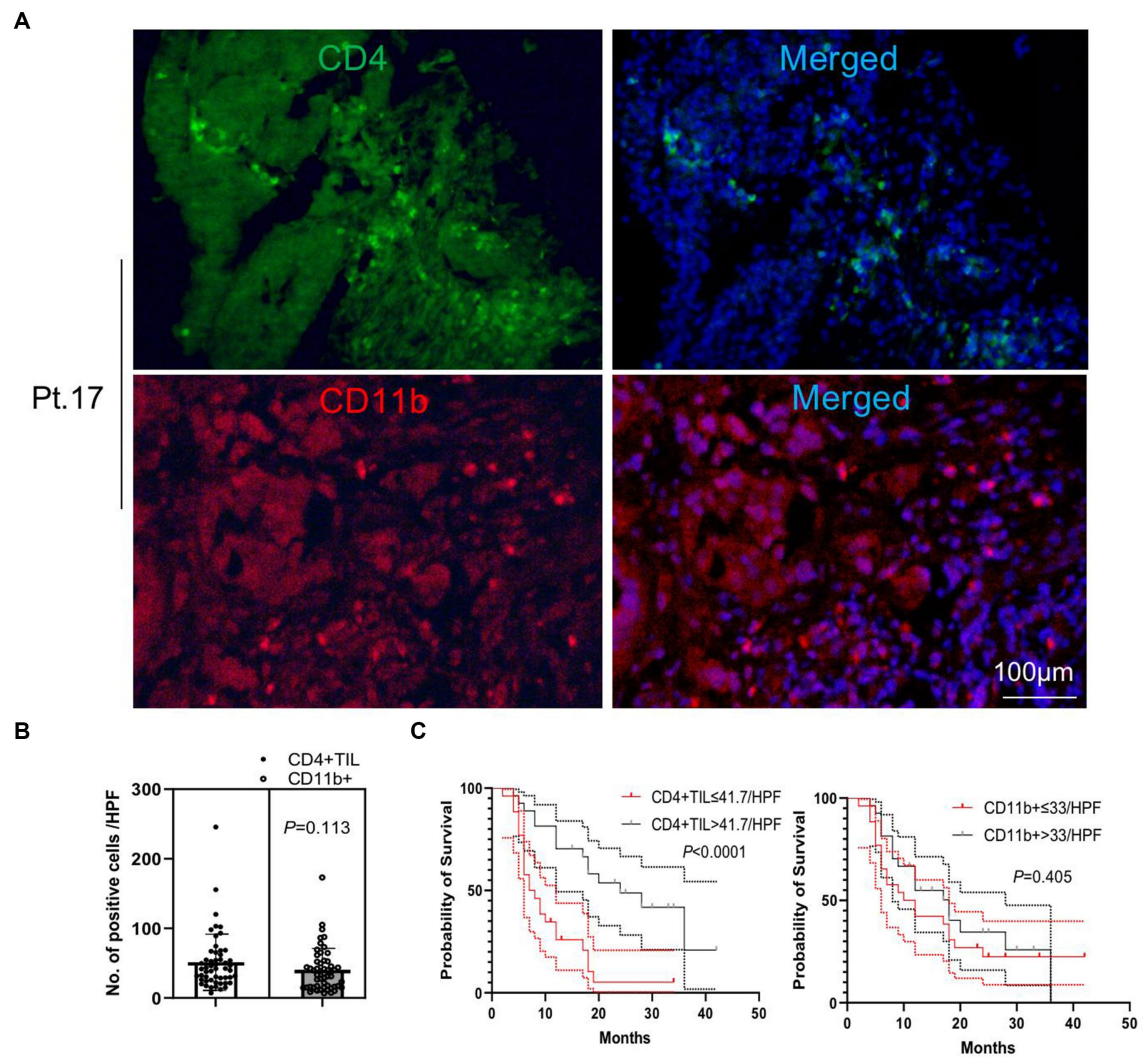
Analysis of tumor-infiltrating CD4+ T cells and CD11b + cells. **(A)** Representative images of immunofluorescence staining of CD4+ tumor-infiltrating lymphocytes (TILs) and tumor-infiltrating CD11b + cells (CD4 in green, CD11b in red, and DAPI in blue). **(B)** Quantitative analysis of CD4+ TILs and tumor-infiltrating CD11b + cells shows that there was no significant difference between the numbers of CD4+ TILs and CD11b + cells (*p* = 0.113). **(C)** Patients with CD4+ TILs higher than 41.7 per HPF experienced an extended overall survival compared to patients with CD4+ TILs lower than 41.7 per HPF (*p* < 0.0001). There was no overall survival difference between patients with tumor-infiltrating CD11b + higher and lower than 33 per HPF (*p* = 0.405). HPF, high power field. Survival comparisons between groups were calculated using the log-rank (Mantel-Cox) test. An unpaired *t*-test was used to analyze the statistical significance between the two groups.

We performed immunofluorescence staining of tumor-infiltrating lymphocytes (TILs) and tumor-infiltrating CD33+ myeloid-derived suppressor cells (MDSCs) to explore their tumor infiltration feature (Figures 2, 3). We found that there were significantly more CD8+ TIL than tumor-infiltrating CD33+ MDSCs cells (*p* = 0.009) (Figures 2A,B). We also found that patients with higher than 46 CD8+ TIL per HPF and patients with lower than 32 tumor-infiltrating CD33+ MDSCs per HPF showed significantly longer overall survival (*p* = 0.011 and *p* = 0.018, respectively) (Figure 2C). Furthermore, quantitative analysis of CD4+ TIL and tumor-infiltrating CD11b + cells show that there was no significant difference between CD4 + TIL and tumor-infiltrating CD11b + cells (*p* = 0.113) (Figures 3A,B). Importantly, patients with higher than 41.7 CD4+ TIL per HPF experienced a more extended overall survival (*p* < 0.0001), and there was no overall survival difference between patients with higher than 33 tumor-infiltrating CD11b + per HPF (*p* = 0.405) and those with lower than 33 CD11b+/HPF (Figure 3C). These results indicated that the numbers of CD8+ and CD4+ tumor-infiltrating lymphocytes were positively associated with prolonged overall survival in patients. Thus, it will be of great interest to study and develop approaches that aim to enhance anti-tumor activity by improving CD8+ and CD4+ T cell infiltration. The number of tumor-infiltrating CD33+ MDSCs was negatively associated with patient overall survival, and therefore downregulating CD33+ MDSCs infiltration is preferred to promote an anti-tumor response.

### Tumor CD8+ and CD4+ T cell infiltrations were significantly associated with tumor size, tumor pathology, and peripheral lymphocyte percentages

**Figure 4:**
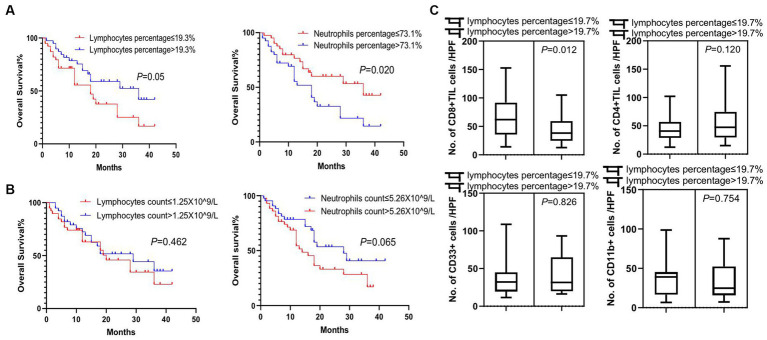
Survival analysis of peripheral white blood cells and association feature of peripheral and tumor-infiltrating immune cells. **(A)** Significant difference in overall survival was observed in patients with lymphocyte percentage of peripheral blood higher than 19.3% or neutrophil percentage lower than 73.1% (*p* = 0.05 and *p* = 0.02, respectively). **(B)** Lymphocyte and neutrophil counts in the peripheral blood of patients were not prognostic factors for the overall survival of patients. **(C)** There was a significant increase in tumor-infiltrating CD8+ T cells (CD8 + TIL) in patients with lower lymphocyte percentages in peripheral blood (*p* = 0.012), while no difference was detected in tumor-infiltrating CD4+ T cells (CD4+ TIL), CD33+ MDSCs, and CD11b + cells between these two groups of patients (*p* = 0.120, *p* = 0.826, and *p* = 0.754, respectively). Survival comparisons between groups were calculated using the log-rank (Mantel-Cox) test. An unpaired *t*-test was used to analyze the statistical significance between the two groups.

To explore the clinical factors that are related to tumor lymphocytes and MDSCs infiltration, we next determined the association feature of peripheral and tumor-infiltrating immune cells based on different clinical factors. We found that patients with a peripheral lymphocyte percentage higher than 19.3% or a neutrophil percentage lower than 73.1% lived significantly longer (*p* = 0.05 and *p* = 0.02, respectively) (Figure 4A). However, peripheral lymphocyte and neutrophil counts were not significant prognostic factors for the overall survival in patients (Figure 4B). We next analyzed tumor lymphocyte infiltration in patients with high and low peripheral lymphocyte percentages. We found that there was a significant increase of tumor-infiltrating CD8+ T cells in patients with lower peripheral lymphocyte percentages when compared to patients with higher peripheral lymphocyte percentages (*p* = 0.012), while there was no difference in terms of tumor-infiltrating CD4+ T cells, CD33+ cells, and CD11b + cells in these two groups (*p* = 0.120, *p* = 0.826, and *p* = 0.754, respectively) (Figure 4C). Although there were no significant correlations between tumor size, peripheral lymphocyte count, peripheral lymphocyte percentage, and tumor-infiltrating CD4+ and CD8+ T cells, trends of correlation were still observed in this relatively small population study (Supplementary Figure S2). Future studies of larger populations are needed to confirm these results.

**Figure 5:**
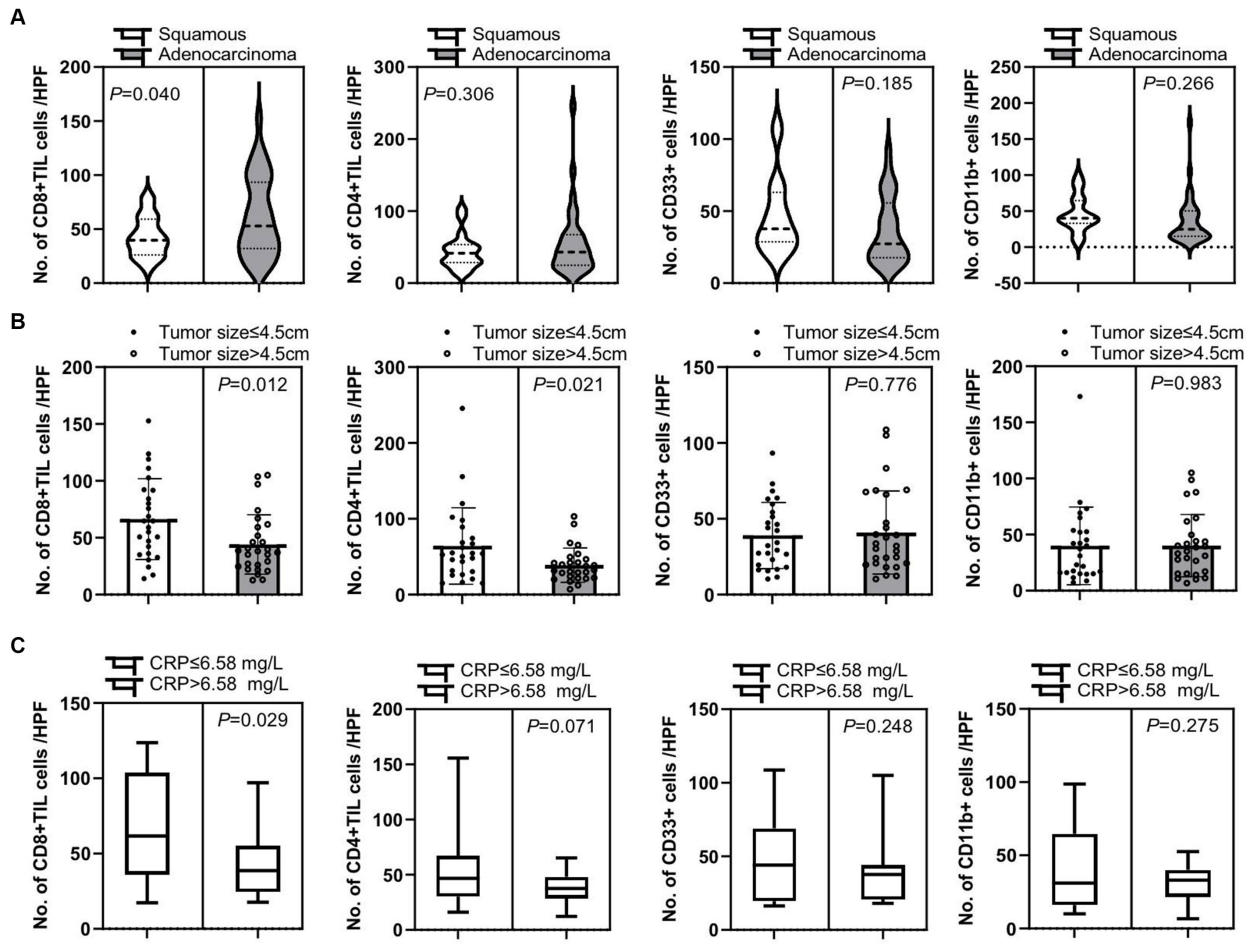
Correlation of tumor-infiltrating immune cells and clinical characteristic factors. **(A)** Patients with adenocarcinoma had a significant increase in tumor-infiltrating CD8+ T cells compared to patients with squamous cell carcinoma (*p* = 0.040), while there is no significant difference in numbers of tumor-infiltrating CD4+ T cells (CD4+ TIL), CD33+ MDSCs, and CD11b + cells between these two groups of patients (*p* = 0.306, *p* = 0.185, and *p* = 0.266, respectively). **(B)** There were significantly increased tumor CD8+ T cell and CD4+ T cell infiltrations in patients with smaller tumor sizes of below 4.5 cm than in patients with larger tumor sizes of over 4.5 cm (*p* = 0.012 and *p* = 0.021, respectively). There was no significant difference in tumor CD33+ MDSCs and CD11b + cell infiltration in these two cohorts of patients (*p* = 0.776 and *p* = 0.983, respectively). **(C)** Patients with C-reactive protein (CRP) expression below 6.58 mg/L in peripheral blood had a significant increase in tumor-infiltrating CD8+ T cells than patients with CRP expression over 6.58 mg/L in peripheral blood (*p* = 0.029), while there is no significant difference in tumor-infiltrating CD4+ T cells, CD33+ MDSCs, and CD11b + cells between these two groups of patients (*p* = 0.071, *p* = 0.248, and *p* = 0.275, respectively). An unpaired *t*-test was used to analyze the statistical significance between the two groups.

Additionally, we confirmed that there were several clinical factors that affected tumor immune cell infiltration. First, patients with adenocarcinoma had a significant increase of tumor-infiltrating CD8+ T cells compared to patients with squamous cell carcinoma (*p* = 0.040), while there was no significant difference in tumor-infiltrating CD4+ T cells, CD33+ MDSCs, and CD11b + cells between these two groups (*p* = 0.306, *p* = 0.185, and *p* = 0.266, respectively) (Figure 5A). Second, there was an significantly elevated infiltration of tumor CD8+ T cells and CD4+ T cells in patients with tumor size below 4.5 cm compared to those with larger tumor size of over 4.5 cm (*p* = 0.012 and *p* = 0.021, respectively), but no difference in tumor CD33+ MDSCs and CD11b + cell infiltration was detected between these two cohorts of patients (*p* = 0.776 and *p* = 0.983, respectively) (Figure 5B). Third, patients with C-reactive protein (CRP) expression below 6.58 mg/L in peripheral blood had a significant increase in tumor-infiltrating CD8+ T cells than patients with CRP expression over 6.58 mg/L in peripheral blood (*p* = 0.029), while there was no significant difference in tumor-infiltrating CD4+ T cells, CD33+ MDSCs, and CD11b + cells between these two groups (*p* = 0.071, *p* = 0.248, and *p* = 0.275, respectively) (Figure 5C).

We further compared tumor-infiltrating CD8+ T cells and CD11b + cells, CD8+ T and CD4 + T cells, CD4 + T and CD33 + MDSCs, and CD11b + and CD33 + MDSCs, and we show that there was no significant difference in CD4+ helper T and CD8+ cytotoxic T cell infiltration in this set of NSCLC patients. We also found a significant difference in CD8+ T cell infiltration compared to infiltrated CD11b + cells (*p* = 0.017) (Supplementary Figure S3). These results suggest that CD8+ cytotoxic T cell infiltration was remarkably more than CD11b + cells, implying that cytotoxic T cells may be more dominant than CD11b + neutrophils or macrophages in playing anti-tumor response in these newly diagnosed stage III/IV NSCLC patients. Further molecular studies on why and how this tumor microenvironment is featured are in dire need.

## Discussion

While PD-1 immune checkpoint inhibitor therapy in metastatic non-small-cell lung cancer (NSCLC) has shown some therapeutic potential (24, 25), most patients do not respond to or are resistant to PD-1 antibodies. Even when applying a combination treatment of PD-1 inhibitors and first-line platinum-based chemotherapy, most patients continue to experience cancer progression within 12 months due to an absence of the activated tumor-specific T cells (25, 26). To address this challenge, researchers began to explore the clinical effects of autologous T-cell adoptive cell therapy (ACT) using TILs. A recent study provides important evidence that TIL therapy has significant clinical benefits for NSCLC patients with resistance to PD-1 immune checkpoint blocking therapy and that 7 out of 16 patients showed objective clinical response (5). This result suggests that autologous TIL therapy is safe and has a promising anti-tumor effect, contributing to the development of novel therapies for metastatic NSCLC. However, the production of TILs requires obtaining sufficient fresh tumor tissues from patients, which is difficult for late-stage NSCLC patients as they usually have limited opportunities for tumor resection. Therefore, it is of great interest to explore the features of natural TIL infiltration in advanced NSCLC patients. This endeavor will not only provide information for precise inclusion criteria of TIL therapy for metastatic NSCLC patients but also pave the way for future studies on TIL infiltration manipulation. Moreover, exploring the detailed features of NSCLC patients who are most likely to respond to TIL therapy is urgently needed.

In the present study, we first explored the prognostic significance of both peripheral and tumor-infiltrating lymphocytes in newly diagnosed stage III/IV NSCLC patients. We confirmed that patients with high tumor CD4+ and CD8+ T cell infiltration survived significantly longer than those with low tumor CD4+ and CD8+ T cell infiltration. High tumor CD33+ MDSC infiltration is associated with worse OS compared to low tumor CD33+ MDSC infiltration. Importantly, we revealed that high tumor CD8+ T cell infiltration was significantly associated with low peripheral lymphocyte count, low serum CRP expression, smaller tumor size, and better tumor pathology. These results indicated that patients with different clinical factors experience different grades of tumor immune cell infiltration. Patients with low peripheral lymphocyte count, low serum CRP expression, small tumor size, and tumor pathology of adenocarcinoma exhibited high TIL infiltration, making them the preferred population for TIL therapy. In addition, we confirmed that TIL infiltration was an important factor for prolonged survival in patients, which is consistent with several previous studies (27–30). Furthermore, we showed that high CD8+ TIL infiltration was correlated with a low peripheral lymphocyte percentage, suggesting that there may be more CD8+ T cells migrating into tumor tissues or other tissues in these patients. At the same time, we showed that a high peripheral lymphocyte percentage is associated with a longer overall survival, implying that the peripheral lymphocyte percentage may be related to a particular TIL cell sub-type playing the strongest anti-tumor effect. However, further studies are needed to delineate the connection and the molecular mechanisms of high peripheral lymphocyte percentage regulating anti-tumor response caused by TILs. Moreover, a shortcoming of the present study was that there was no data analysis of detailed T cell sub-typing of peripheral lymphocytes to further dissect the connection between peripheral and tumor-infiltrating lymphocytes.

It is worth noting that the results of the current study present several future research directions. First, it will be of interest to investigate what underlying molecular mechanisms regulate TIL infiltration. Second, future studies engineering TILs to improve their anti-tumor activity by either ablating or overexpressing certain biomarkers are warranted. Third, it is important to reveal whether there is any T cell exhaustion of the TILs and what their special features are in the tumor tissue. Fourth, it is also important to determine and compare the peculiarity of the TILs across different types of cancer. Finally, future studies are needed to understand which patient populations are appropriate for TIL treatment based on the extent of TIL infiltration and what is the best treatment window for patients to receive TIL therapy in the clinic.

## Conclusion

In conclusion, we reported the prognostic significance of TILs in newly diagnosed stage III/IV NSCLC patients and believe that these results will pave the way for further developing TIL immunotherapy for cancer treatment.

## Data availability statement

The raw data supporting the conclusions of this article will be made available by the authors, without undue reservation.

## Ethics statement

The studies involving humans were approved by Ethics committee of Tianjin Beichen hospital. The studies were conducted in accordance with the local legislation and institutional requirements. The participants provided their written informed consent to participate in this study.

## Author contributions

FL: Conceptualization, Data curation, Formal analysis, Funding acquisition, Project administration, Resources, Writing – original draft, Writing – review & editing. CT: Data curation, Funding acquisition, Investigation, Validation, Writing – review & editing. YW: Data curation, Funding acquisition, Investigation, Resources, Writing – review & editing. HW: Data curation, Software, Validation, Writing – review & editing. MJ: Data curation, Methodology, Software, Writing – review & editing. XD: Resources, Supervision, Visualization, Writing – review & editing. JY: Data curation, Investigation, Resources, Writing – review & editing. XY: Methodology, Resources, Software, Writing – review & editing. HY: Conceptualization, Supervision, Visualization, Writing – review & editing.

## Supplementary material

SUPPLEMENTARY FIGURE S1 Univariate analysis of clinical factors of the studied patients. **(A)** White blood cell count, **(B)** CA153, **(C)** neutrophils count to lymphocyte count, **(D)** CEA, **(E)** with and without distant metastasis, and **(F)** with and without pleural effusion were not prognostic factors for the overall survival (OS) of the patients. Survival comparisons between groups were calculated using the Log-rank (Mantel-Cox) Test.

SUPPLEMENTARY FIGURE S2 Correlation analysis of peripheral and tumor-infiltrating lymphocytes. There was no significant correlation between tumor size and **(A)** tumor-infiltrating CD4+ T cells and **(B)** CD8+ T cells, **(C)** peripheral lymphocyte counts and tumor-infiltrating CD4+T cells and **(D)** CD8+ T cells, **(E)** peripheral lymphocyte percentages and tumor infiltrating CD4+ T cells, and **(F)** CD8+ T cells.

SUPPLEMENTARY FIGURE S3 Comparisons of tumor infiltrated CD8+ and CD4+ T cells **(A)**, CD8+ T cells and CD11b+ cells **(B)**, CD4+ T cells and CD33+ MDSCs **(C)**, and CD11b+ and CD33+ MDSCs **(D)**.
