# Supplementary material for: Prognostic significance of peripheral and tumor-infiltrating lymphocytes in newly diagnosed stage III/IV non-small-cell lung cancer
Source: Front Med (Lausanne). 2024 May 22;11:1349178. doi: 10.3389/fmed.2024.1349178 (PMC11150824; doi:10.3389/fmed.2024.1349178)
Supplement: Supplementary file 1 [file Table_1.doc]

**Supplemental Table 1 Survival comparisons between subgroups of different clinical characters**

| **Clinical characters** | **Median Survival (Months, n=86)** | **95% CI of ratio** | ***P* value** |
| --- | --- | --- | --- |
| White blood cell count≤6.84X10^9^/L | 19 | 0.5893-1.891 | 0.415 |
| White blood cell count>6.84X10^9^/L | 18 |  |  |
| Neutrophils percentage≤73.1% | 36 | 1.068-3.746 | **0.020** |
| Neutrophils percentage>73.1% | 18 |  |  |
| Neutrophils count≤5.26X10^9^/L | 28 | 1.106-3.616 | 0.065 |
| Neutrophils count>5.26X10^9^/L | 14 |  |  |
| Lymphocytes percentage≤19.3% | 18 | 0.2626-0.9520 | **0.05** |
| Lymphocytes percentage>19.3% | 36 |  |  |
| Lymphocytes count≤1.25X10^9^/L | 20 | 0.3648-1.304 | 0.497 |
| Lymphocytes count>1.25X10^9^/L | 29 |  |  |
| CRP≤8.29mg/L | 29 | 1.130 -4.404 | **0.011** |
| CRP>8.29mg/L | 13 |  |  |
| Neutrophils count/lymphocyte count ≤3.76 | 36 | 1.050-3.808 | 0.064 |
| Neutrophils count/lymphocyte count >3.76 | 18 |  |  |
| CA153≤11.3U/ml | 28 | 0.3368 -1.197 | 0.160 |
| CA153>11.3U/ml | 15 |  |  |
| CEA≤6.98ng/mL | 20 | 0.6461 -2.142 | 0.324 |
| CEA>6.98ng/mL | 17 |  |  |

CEA,carcinoembryonic antigen; CA153,cancer antigen 153 ;CRP,C Reactive Protein;SQ,Squamous;AD,adenocarcinoma
