# Supplementary figures and images for: Prognostic significance of peripheral and tumor-infiltrating lymphocytes in newly diagnosed stage III/IV non-small-cell lung cancer
Source: Front Med (Lausanne). 2024 May 22;11:1349178. doi: 10.3389/fmed.2024.1349178 (PMC11150824; doi:10.3389/fmed.2024.1349178)

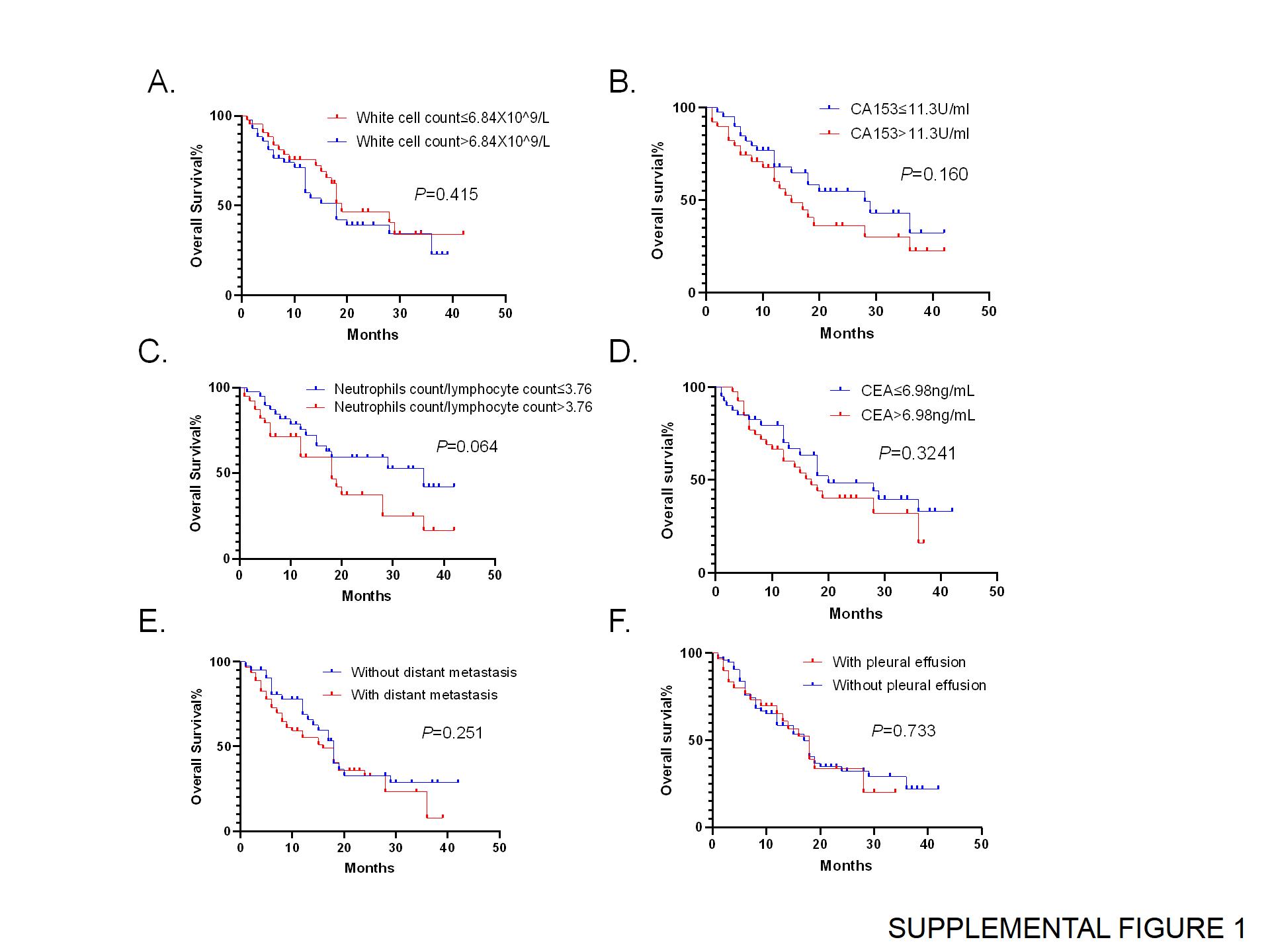

Supplement: SUPPLEMENTARY FIGURE S1 — Univariate analysis of clinical factors of the studied patients. (A) White blood cell count, (B) CA153, (C) neutrophils count to lymphocyte count, (D) CEA, (E) with and without distant metastasis, and (F) with and without pleural effusion were not prognostic factors for the overall survival (OS) of the patients. Survival comparisons between groups were calculated using the Log-rank (Mantel-Cox) Test. [file Image_1.JPEG]

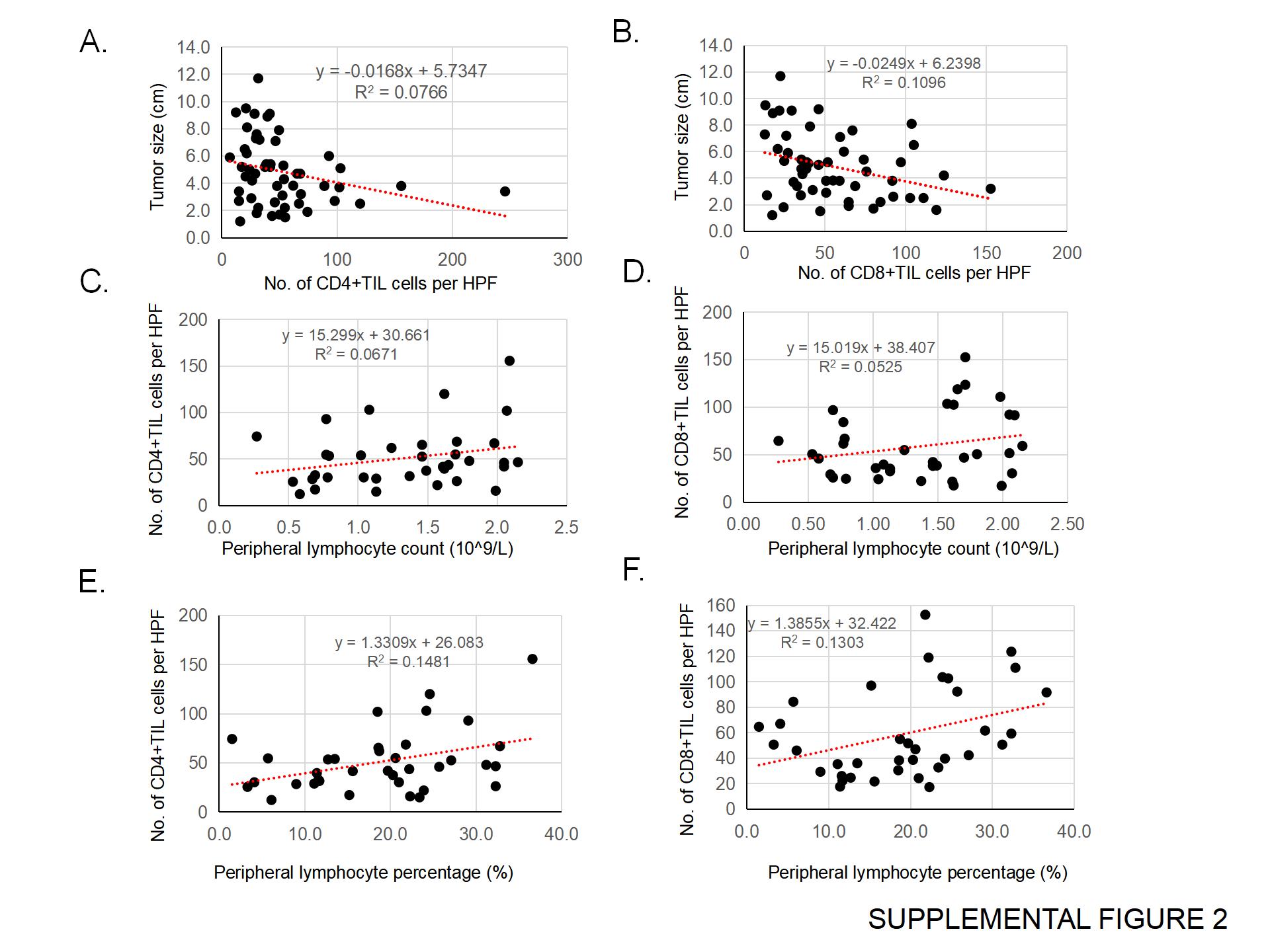

Supplement: SUPPLEMENTARY FIGURE S2 — Correlation analysis of peripheral and tumor-infiltrating lymphocytes. There was no significant correlation between tumor size and (A) tumor-infiltrating CD4+ T cells and (B) CD8+ T cells, (C) peripheral lymphocyte counts and tumor-infiltrating CD4+T cells and (D) CD8+ T cells, (E) peripheral lymphocyte percentages and tumor infiltrating CD4+ T cells, and (F) CD8+ T cells. [file Image_2.JPEG]

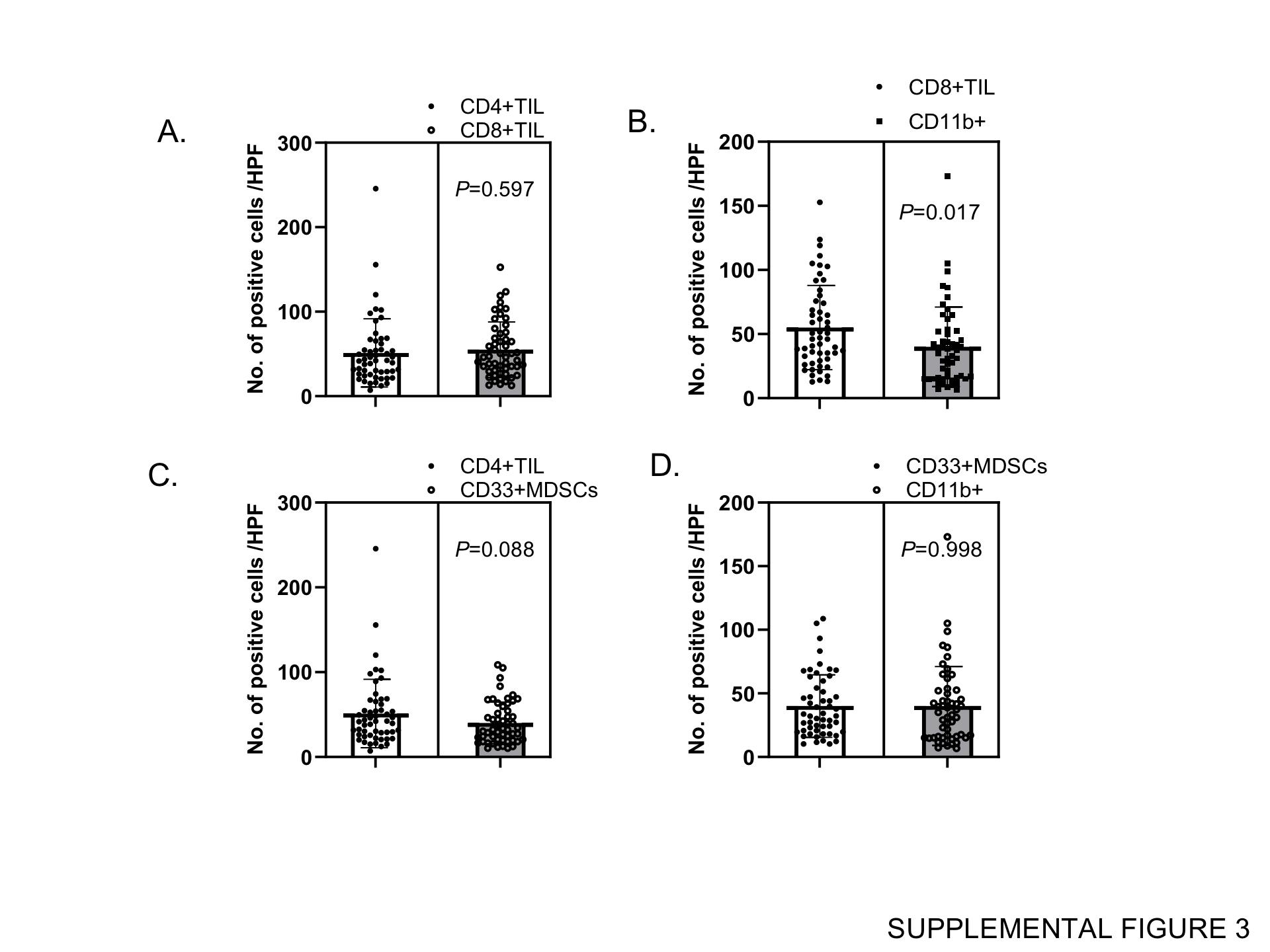

Supplement: SUPPLEMENTARY FIGURE S3 — Comparisons of tumor infiltrated CD8+ and CD4+ T cells (A), CD8+ T cells and CD11b+ cells (B), CD4+ T cells and CD33+ MDSCs (C), and CD11b+ and CD33+ MDSCs (D). [file Image_3.JPEG]
